# MiR-27b-3p Inhibition Enhances Browning of Epididymal Fat in High-Fat Diet Induced Obese Mice

**DOI:** 10.3389/fendo.2019.00038

**Published:** 2019-02-04

**Authors:** Jing Yu, Yifan Lv, Fengliang Wang, Xiaocen Kong, Wenjuan Di, Juan Liu, Yunlu Sheng, Shan Lv, Guoxian Ding

**Affiliations:** ^1^Division of Geriatric Endocrinology, The First Affiliated Hospital of Nanjing Medical University, Nanjing, China; ^2^Department of Breast Surgery, The Affiliated Obstetrics and Gynaecology Hospital of Nanjing Medical University, Nanjing Maternity and Child Health Care Hospital, Nanjing, China

**Keywords:** miR-27b-3p, high-fat diet, fat browning, central obesity, epididymal fat

## Abstract

**Objective:** Long-term dysregulation of energy balance is the key component of the obesity epidemic. Given the harm of central obesity and the discovery that beige cells appear within white adipose tissue (WAT), enhancing the energy-expending or “browning” ability of visceral adipose tissue (VAT) has become of therapeutic interest. In this study, we focused on the regulating role of microRNA (miRNA)-27b-3p in mice epididymal white adipose tissue (eWAT) browning.

**Methods:** High-fat diet (HFD) induced obese mice model was constructed. Expression of miR-27b-3p and Ucp1 in eWAT was measured during the course of HFD. Through tail vein injection of antimiR-27b-3p, miR-27b-3p expression was inhibited to analyze the potential role of miR-27b-3p in fat browning and metabolism.

**Results:** miR-27b-3p was predominantly expressed in eWAT and browning ability of eWAT in HFD induced obese mice was impaired. Inhibition of miR-27b-3p enhanced browning capacity of eWAT in mice fed an HFD and led to weight loss and insulin sensitivity improvement.

**Conclusions:** High expression of miR-27b-3p in eWAT inhibits browning ability and leads to visceral fat accumulation. It is suggested miR-27b-3p may become a potential therapeutic option for visceral obesity and its associated diseases.

## Introduction

Obesity is an epidemic with more than 300 million people being clinically obese worldwide. Although obesity is a risk factor for insulin resistance and type two diabetes, and a significant risk factor for cardiovascular diseases (CVDs), not every obese patient is insulin resistant or at high risk of diabetes and CVDs (1). The subgroup of individuals with a selective excess of intra-abdominal, or visceral adipose tissue (VAT) is at substantially higher risk of being characterized by insulin resistance, hyperglycemia, dyslipidemia and CVDs (2, 3). Visceral obesity may be a unifying pathological state predisposing people to these various comorbidities.

The pathogenesis of obesity is extremely complex and is not completely understood, but the key component of the obesity epidemic is long-term dysregulation of energy balance, comprising increased energy intake and/or reduced energy expenditure. As a result, impacting energy balance in humans would be of significant clinical importance. The discovery that white pre-adipocytes can be differentiated into beige adipocytes *in vitro* (4, 5) has renewed scientific interest in white adipose tissue (WAT) and its role in energy expenditure. With the induction of beige adipocytes in WAT, the expression of uncoupling protein 1 (Ucp1) increases. Activated Ucp1 transfers energy for the phosphorylation of ADP to ATP by uncoupling the proton gradient (6), which can increase energy expenditure, glucose and fatty acid oxidation, and improve insulin sensitivity (7–9). Thus, browning of WAT is an appealing therapeutic target for obesity and its associated diseases.

Over the past few years, the role of microRNAs (miRNAs) in the regulation of different biological processes has become evident. Accumulating evidence have suggested miRNAs as a new layer of regulatory mechanism for brown adipogenesis, which provides new insights for prevention and treatment of metabolic diseases (10, 11). MiRNAs such as miR-193b-365 cluster, miR-328, miR-378, miR-33b/c, miR-455, and miR-32 are activators of brown adipogenesis, while miR-34a, miR-133, and miR-155 are inhibitors of brown adipogenesis (11). Our previous study demonstrated that glucocorticoids transcriptionally regulate expression of miR-27b (now miR-27b-3p) and miR-19b, promoting body fat accumulation by suppressing the browning of mouse WAT (12, 13). We also verified the role of miR-27b-3p in regulating browning of human visceral adipocytes (14). However, so far there has no report about the role of miR-27b-3p in regulating browning of visceral fat obtained from high fat diet (HFD) induced obese rodents.

Here, we generated an HFD induced obesity mouse model to confirm the effects of miR-27b-3p on browning of epididymal white adipose tissue (eWAT) which was considered as visceral fat tissue for their similar characteristics. We found that miR-27b-3p was predominantly expressed in eWAT and browning ability of eWAT in HFD induced obese mice was impaired. Moreover, miR-27b-3p inhibition enhanced browning capacity of eWAT in mice fed an HFD and led to weight loss and insulin sensitivity improvement. Taken together, our results demonstrate that high expression of miR-27b-3p in eWAT inhibits browning ability and leads to visceral fat accumulation. It is suggested miR-27b-3p may become a potential therapeutic option for visceral obesity and its associated diseases.

## Materials and Methods

### Study Approval

Animal welfare and experimental procedures were carried out strictly in accordance with National Research Council guidelines for the care and use of laboratory animals (1996) and were reviewed and approved by the Model Animal Research Center (Nanjing University) Institutional Animal Care and Use Committee.

### Animals

Male C57BL/6J mice were purchased from the Model Animal Research Center of Nanjing University (Nanjing, China). For all experiments, mice were maintained on a normal 12:12-h light-dark cycle and provided regular mouse chow and water *ad libitum* at an Association for Assessment and Accreditation of Laboratory Animal Care (AAALAC)-accredited specific pathogen-free facility.

Three-week old mice were randomly assigned to 2 groups (*n* = 12–25/group) and provided *ad libitum* access to high fat diet (HFD, 60% calories as fat) or normal chow fat (NCD, 10% calories as fat) for 13 weeks. Food intake and body weight were measured weekly. When characteristics of adipose tissue were studied during the course of HFD, mice were sacrificed at the time points of 1st, 2nd, 4th, 6th, 8th, and 10th week after starting with an HFD. At the time of sacrifice, adipose tissues were weighed, then frozen in liquid nitrogen and stored at −80°C.

### *In vivo* Gene Delivery—Tail Vein Injection With Lentiviruses

Lentiviruses encoding the anti-sense to miR-27b-3p (antimiR-27b-3p) with the sequence of GCAGAACTTAGCCACTGTGAA or a scrambled control (scr-miR) were obtained from Genechem Inc (Shanghai, China). For *in vivo* gene delivery, mice were injected with 6 × 10^7^ transducing units (TU)/mouse of each lentivirus in 100 μl PBS through the tail vein 2 times in total (one time per wk) after treatment with high fat diet for 13 weeks. Mice were sacrificed on the 14th day after the lentiviral injection.

### Whole-Body Energy Metabolism

Whole-body energy metabolism was evaluated using the Comprehensive Lab Animal Monitoring System (CLAMS, Columbus Instruments, OH, USA). Mice were acclimated in individual metabolic chambers with free access to food and water, allowing them to acclimatize for 2 days before starting the experiments. Subsequently, CO_2_ and O_2_ levels were monitored every 30 min for each mouse over a period of 3 days. The sensor was calibrated against a standard gas mix containing defined quantities of O_2_ and CO_2_. The heat index was calculated using the following formula: (3.815 + 1.232 × VCO_2_/VO_2_) × VO_2_. Spontaneous locomotor activity was recorded with OPTO-M3 Activity Application Device (Columbus Instruments). Total activity and food intake were measured at regular intervals.

### Oral Glucose Tolerance Test

Oral glucose tolerance test (oGTT) was performed following a 16-h fast by measuring blood glucose on glucose strips and with an Accu-check glucometer (Roche, Basel, Switzerland) at baseline (0 min) and at 15, 30, 60, and 120 min after an oral bolus dose of glucose at 2 g/kg body weight. Blood was obtained from a tail nick.

### Insulin Tolerance Test

For the insulin tolerance test (ITT), mice were fasted for 6 h prior to obtain baseline blood glucose reading (0 min), followed by an intraperitoneal injection of insulin (Eli Lilly, IN, USA) at 0.5 U/kg body weight. Blood glucose was then measured at 15, 30, 60 and 120 min after insulin injection. Blood was obtained from a tail nick, and blood glucose was also measured with glucose strips and an Accu-check glucometer (Roche).

### Adipose Tissue Histology and Immunohistochemistry

Epididymal, inguinal subcutaneous and retroperitoneal fat tissues were fixed in 10% formalin, processed and paraffin-embedded. Multiple sections (5 μm) were prepared and stained with hematoxylin and eosin (H&E) for general morphological observation. For immunocytochemistry staining, the sections of VAT were incubated with anti-UCP1 antibody (Abcam, MA, USA) for 30 min at room temperature. The signals were detected using a biotinylated goat anti-rabbit secondary antibody (Vector Laboratories, CA, USA) in combination with the ABC kit (Vector Laboratories) and DAB substrate (Vector Laboratories). Samples were visualized using a Nikon Eclipse 80i upright microscope (Nikon, Tokyo, Japan).

### Cell Culture and Transfection

Isolation of primary stromal vascular fraction (SVF) cells from brown adipose tissue (BAT)—Mouse BAT was isolated from 3-week-old male C57BL/6J mice. The tissue was minced and digested with 10 mg/ml type II collagenase solution (Gibco, Grand Island, New York). The resulting cell suspension was then filtered through a 50-μm nylon mesh and the filtrate centrifuged at low speed. The stromal vascular fraction (SVF) cells in the pellet were re-suspended, plated in 12 multiwell culture plates and grown in DMEM containing 10% fetal bovine serum until confluence. For adipocytes differentiation, the confluent SVF cells were differentiated for 4 days in a differentiation medium (DMEM medium containing 10% fetal bovine serum, 0.5 mM 3-isobutyl-1-methylxanthine, 1 μM dexamethasone and 20 nM insulin) (Sigma-Aldrich, MO, USA). SVF cells from BAT were induced by treating with 20 nM insulin for another 2 days and collected at Day 6. The medium was changed every 2 days.

Cell transfection—AntimiR-27b-3p transfection—SVF cells isolated from BAT were cultured in growth medium till 80% confluence, which then were transfected with 2 nM locked nucleic acid (LNA)-modified antimiR-27b-3p oligonucleotides (methylene bridge between the 2′-O and the 4′-C atoms, sequence 5'-GCAGAACTTAGCCACTGTGA-3') (Exiqon, Vedbaek, Denmark) or LNA scrambled miR-27b-3p control (scr-miR). Twenty-four hours after transfection, medium was replaced by fresh growth medium. When the cells reached to complete confluence the adipocyte differentiation was then initiated by replacing the adipocyte differentiation medium and cultured for further 4 days.

### Quantitative Real-Time PCR (qRT-PCR)

Total RNA was isolated from tissues with TRIZOL® Reagent (Invitrogen, CA, USA) according to the manufacturer's instructions. A sample of total RNA (2 μg) was reverse-transcribed with 200 U of Moloney murine leukemia virus reverse transcriptase (M-MLV) in the presence of 0.5 mM deoxynucleotide triphosphate, 25U RNase inhibitor, and 10 mM random hexamer primers (Promega, WI, USA), in a total volume of 25 μL. PCR primers (Table S1) were designed by Primer 5 software (PREMIER Biosoft, CA, USA). Expression level was normalized to that of β-actin. Each quantitative real-time PCR (qRT-PCR) was carried out in triplicate, in a final volume of 25 μL of SYBR Green Real-time PCR Master Mix (Roche).

For miRNA qRT-PCR, a miRNA-specific stem-loop RT primer was hybridized to the miRNA and then reverse transcribed. The RT product was then amplified and monitored in real time using a miRNA-specific upstream primer and the universal downstream primer (Table S1). Expression level was normalized to that of small non-coding RNA U6.

PCR conditions were as follows: 10 min at 95°C, followed by 40 cycles of 15 s at 95°C and 60 s at 60°C. The temperature was increased from 60 to 95°C and the PCR melting curve was made every 1.0°C after the amplification reaction (ABI 7000, StepOnePlus, CA, USA). The mean value of triplicates for each sample was calculated and expressed as the cycle threshold (Ct). Gene expression was then calculated as the ΔCt, the difference between the Ct value of the sample and the Ct value of β-actin or U6, which was used as an internal reference. The relative expression level was evaluated using the comparative delta-delta Ct method (2^−ΔΔCt^).

### Protein Extraction and Western Blotting

Fat tissues were ground by a homogenizer. Total protein was extracted with radioimmunoprecipitation buffer (50 mM Tris-HCl, pH 7.4, 150 mM NaCl, 1% [v/v] Nonidet P-40, 1 mM EDTA, 1 mM NaF, 10 μg/mL aprotinin, 10 μM leupeptin, and 1 mM phenylmethanesulfonyl fluoride (PMSF); Amresco, OH, USA) and allowed to stand on ice for 30 min to permit lysis. After centrifugation at 11,000 rpm for 10 min at 4°C, protein concentration in the supernatant was determined with the BCA assay (Pierce, IL, USA). Aliquots (60 μg) of proteins were separated by 12% sodium dodecyl sulfate (SDS)-polyacrylamide gel electrophoresis and transferred onto a poly (vinylidene difluoride) membrane (Merck Millipore, MA, USA). The membrane was blocked with 1% (w/v) BSA (Thermo Fisher Scientific, MA, USA) in TBST (10 mM Tris-HCl, pH7.8, 150 mM NaCl, and 0.1% TWEEN 20) for 2 h and then incubated with primary polyclonal antibody (anti-UCP1, ab10983, or anti-a-tubulin, ab18251, 1:1000) (Abcam, MA, USA) in TBST containing 1% (w/v) BSA overnight at 4°C. The blots were treated with horseradish peroxidase-conjugated IgG (1:10000; Kirkegaard & Perry Labs Inc., Maryland, USA) in TBST containing 1% (w/v) BSA for 60 min, and the immune complex was detected by using an ECL plus detection kit (Cell Signaling Technology, MA, USA). Bands were quantified by using densitometric image analysis software (Quantity One; Bio-Rad, CA, USA).

### Statistics

Data are presented as the mean ± SEM. Significant differences between two groups were analyzed using two-tailed, unpaired *t*-tests. One-way ANOVA was used for comparison between multiple groups. GraphPad Prism software (Version 6.0c; GraphPad Software Inc., CA, USA) was used for statistical analysis. *p* < 0.05 was considered statistically significant.

## Results

### Characteristics of High-Fat Diet (HFD) Induced Obese Mice

**Figure 1:**
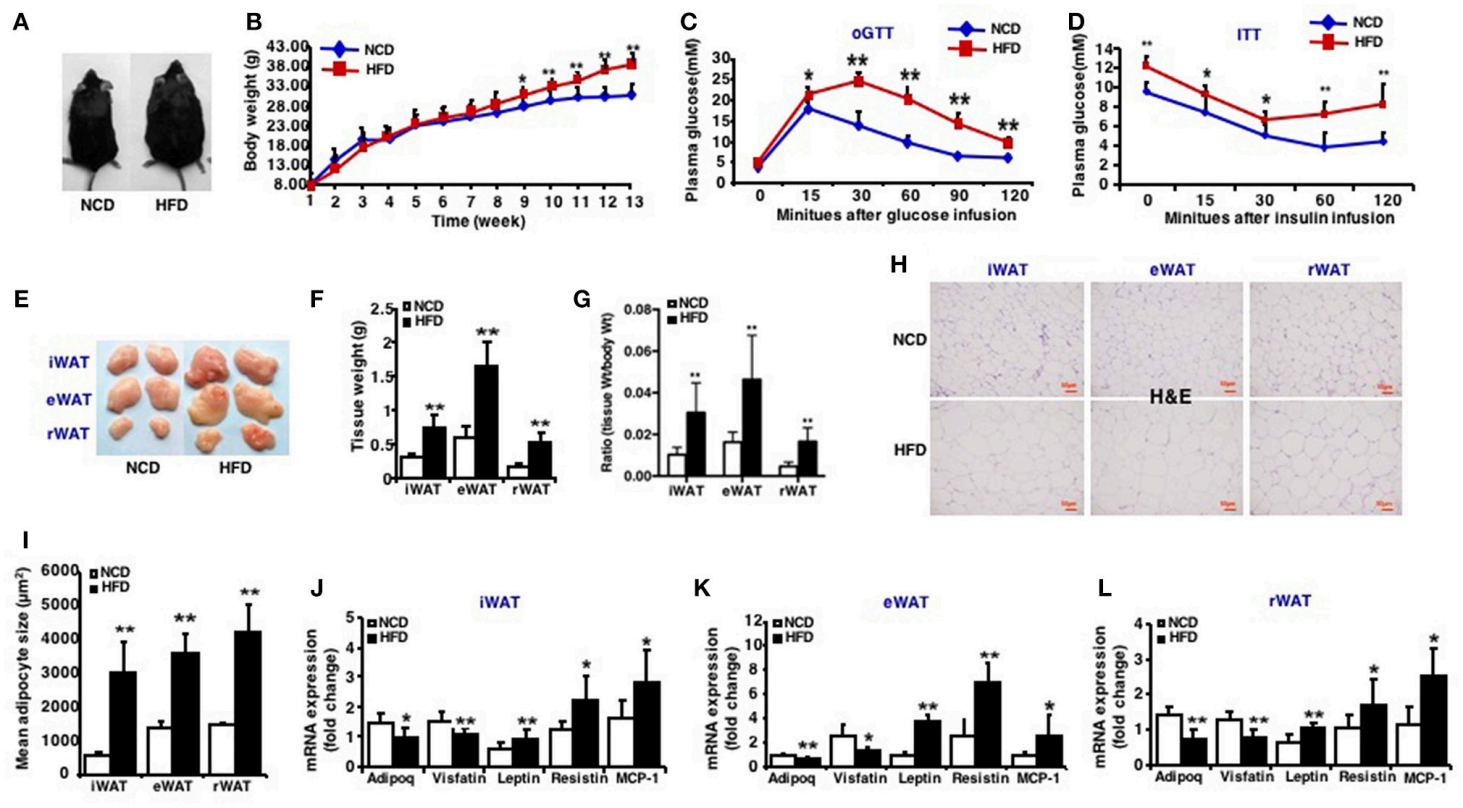
Characteristics of high-fat diet induced obese mice. Mice were fed a high fat diet beginning at 3 weeks of age. Measurements were performed after 13-week feeding. Epididymal (eWAT), inguinal subcutaneous (iWAT) and retroperitoneal white adipose tissue (rWAT) was collected respectively. **(A)** Representative appearance of mice fed with high-fat diet (HFD) and with normal chow diet (NCD). **(B)** Total body weight (*n* = 10–12). **(C)** oGTT, performed after 12 weeks of HFD and mice were fasted for 16 h prior to obtain baseline blood glucose reading (*n* = 6). **(D)** ITT, performed after 13 weeks of HFD and mice were fasted for 6 h prior to obtain baseline blood glucose reading (*n* = 6). **(E)** Representative appearance of iWAT, eWAT, and rWAT. **(F)** Tissue weight (*n* = 10–12). **(G)** Ratio of tissue weight to body weight (*n* = 10–12). **(H)** Representative H&E staining of iWAT, eWAT, and rWAT (*n* = 6). Scale bars: 50 μm. (**I)** Mean adipocyte size was measured under microscope (*n* = 6). **(J–L)** qRT-PCR analysis of adipokines (Adipoq, Visfatin, Leptin, and Resistin) and inflammatory factors (MCP-1) expression of iWAT, eWAT, and rWAT (*n* = 10–12). Data are shown as mean ± SEM. ^*^p < 0.05; ^**^p < 0.01.

The 3-week-old C57BL/6J male mice were given a 13-week high-fat diet intervention, successfully established a high-fat diet-induced obese mouse model (Figure 1A). After 13 weeks of high-fat feeding, mice fed an HFD had significantly increased body mass (Figure 1B) and were glucose intolerant compared to mice fed a normal chow diet (NCD) (Figure 1C). High-fat-fed mice were also insulin resistant, as determined by insulin tolerance tests (Figure 1D). The mice were sacrificed after 13-week high-fat diet and the fat weight of three depots: inguinal WAT (iWAT), epididymal WAT (eWAT), retroperitoneal WAT (rWAT) was measured (Figures 1E,F). The ratio of iWAT, eWAT and rWAT weight to body weight was calculated, respectively (Figure 1G). The size of adipocytes from these fat depots increased markedly after an HFD feeding (Figures 1H,I). The expression of insulin sensitive adipokins such as Adipoq and Visfatin decreased and factors like Leptin, Resistin, and MCP-1 increased in three fat depots of mice fed an HFD (Figures 1J–L). These results displayed the characteristics of HFD induced obese mice and the model was generated successfully.

### miR-27b-3p Is Predominantly Expressed in eWAT Compared With iWAT and rWAT

**Figure 2:**
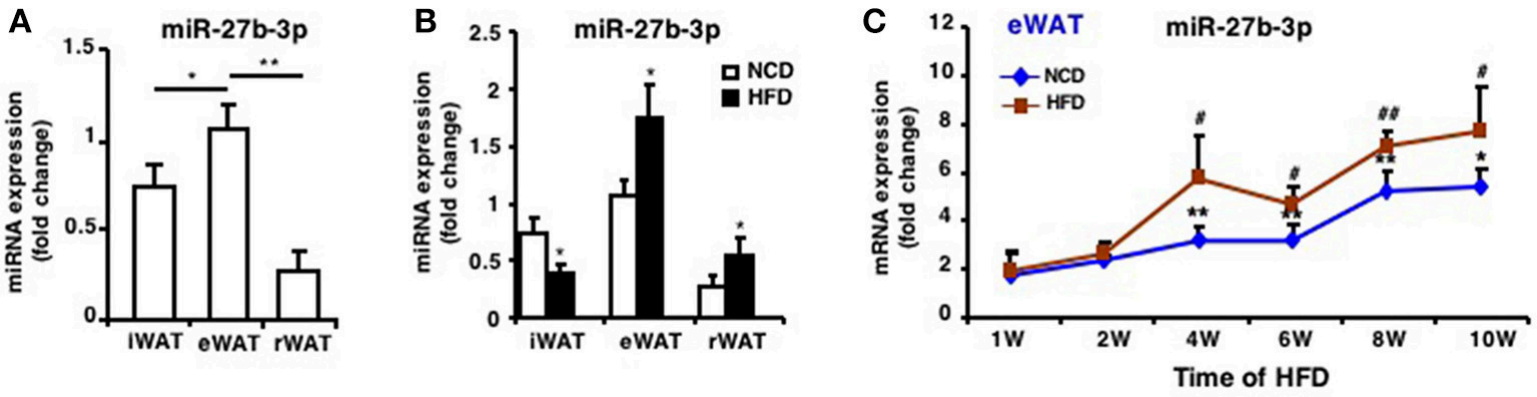
miR-27b-3p is predominantly expressed in eWAT compared with other white adipose tissues. **(A)** qRT-PCR analysis of miR-27b-3p expression in iWAT, eWAT, and rWAT from mice fed a NCD. **(B)** qRT-PCR analysis of miR-27b-3p expression in iWAT, eWAT, and rWAT after 13-week HFD or NCD feeding. **(C)** qRT-PCR analysis of miR-27b-3p expression in eWAT at the 1st, 2nd, 4th, 6th, 8th, and 10th week after HFD. Data are shown as mean ± SEM, *n* = 6. ^*^p < 0.05; ^**^p < 0.01, NCD vs. HFD; ^#^*p* < 0.05; ^##^*p* < 0.01, 2nd, 4th, 6th, 8th, or 10th week vs. 1st week in the HFD group.

In our previous studies, we found miR-27b-3p, under glucocorticoid regulation, inhibited the browning ability of mice iWAT (12) and its role in regulating browning of visceral adipocytes in humans with obesity (14). Before we determined the role of miR-27b-3p in eWAT of mice fed an HFD, expression of miR-27b-3p in three fat depots was analyzed. In mice fed an NCD, miR-27b-3p expression levels in eWAT were significantly higher than in iWAT and rWAT (Figure 2A). Moreover, HFD resulted in a significant increase in the expression of miR-27b-3p in eWAT and rWAT of mice (Figure 2B), and the most outstanding elevation was in eWAT.

To determine the change of miR-27b-3p expression during the course of HFD, we collected eWAT after the mice were fed an HFD for 1, 2, 4, 6, 8, and 10 weeks. With the extension of high-fat feeding duration, expression of miR-27b-3p increased and was consistently higher in eWAT of mice fed an HFD, especially at 4th, 6th, 8th, and 10th week (Figure 2C). These results suggest a relatively more remarkable role of miR-27b-3p in regulating function of eWAT.

### Impaired Browning Ability of eWAT in HFD Induced Obese Mice

**Figure 3:**
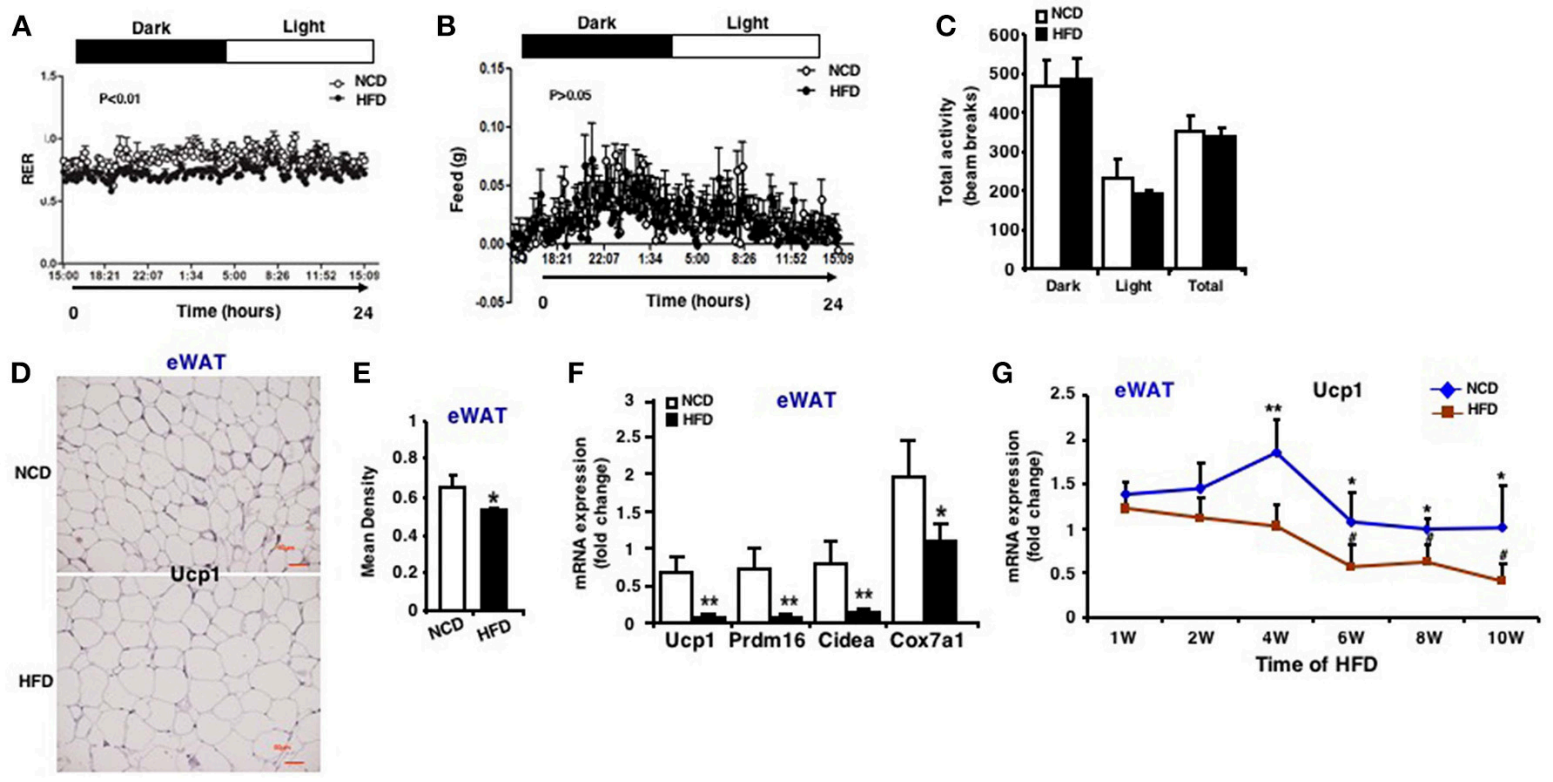
Impaired browning ability of eWAT in HFD induced obese mice. Whole body-energy metabolism analysis was measured after 13-week HFD feeding. **(A)** Respiratory Exchange Rate (RER). **(B)** Food intake. **(C)** Physical activity (*n* = 6). Mice were sacrificed after 13-week HFD feeding. **(D)** Representative immunohistochemical staining for Ucp1 in eWAT (*n* = 6). Scale bars: 50 μm. **(E)** Representative Ucp1 staining intensity designated by mean densitometry of the digital image (*n* = 6). **(F)** qRT-PCR analysis of Ucp1 expression and other brown adipose-selective genes in eWAT (*n* = 10–12). **(G)** qRT-PCR analysis of Ucp1 expression in eWAT at the 1st, 2nd, 4th, 6th, 8th, and 10th week after HFD. Data are shown as mean ± SEM, *n* = 6. ^*^p < 0.05; ^**^p < 0.01, NCD vs. HFD; ^#^*p* < 0.05, 2nd, 4th, 6th, 8th, or 10th week vs. 1st week in the HFD group.

In order to verify the change of browning ability of fat tissues, whole body-energy metabolism analysis was firstly measured and the results revealed that although there was no difference in food intake, physical activity (Figures 3B,C), mice fed an HFD had significantly lower respiratory exchange ratio (RER) during both the active feeding (dark) and fasting phase (light) (Figure 3A), indicating an increased fatty acid oxidation. RER was calculated with the formula VO_2_/VCO_2_ and the oxygen consumption and carbon dioxide production were showed in Figure S1. Quantitative immunohistochemistry analysis revealed that there is less Ucp1-positive cells in eWAT of mice fed an HFD compared to an NCD (Figures 3D,E). In line with this, mRNA expression of Ucp1 decreased by ~7-fold in eWAT of mice fed an HFD (Figure 3F). In addition, mRNA expression of other marker genes of fat browning like Prdm16, Cidea, and Cox7a1 were also decreased (Figure 3F). Similar expression patterns were also seen in iWAT and rWAT (Figure S2).

During the course of HFD, Ucp1 mRNA expression in eWAT of mice fed an HFD was downregulated and decreased with the increasing time of HFD, and the difference between the two groups was more apparent at the 4th, 6th, 8th, and 10th week (Figure 3G). According to the results of miR-27b-3p above, we found that during the HFD process, expression of miR-27b-3p and Ucp1 showed opposite trends. Collectively, these data provide evidence that browning ability of WAT is impaired in response to HFD induced obesity, typically in eWAT. It may probably be related to high level of miR-27b-3p in eWAT.

### Inhibition of miR-27b-3p Improves Browning Ability of eWAT

**Figure 4:**
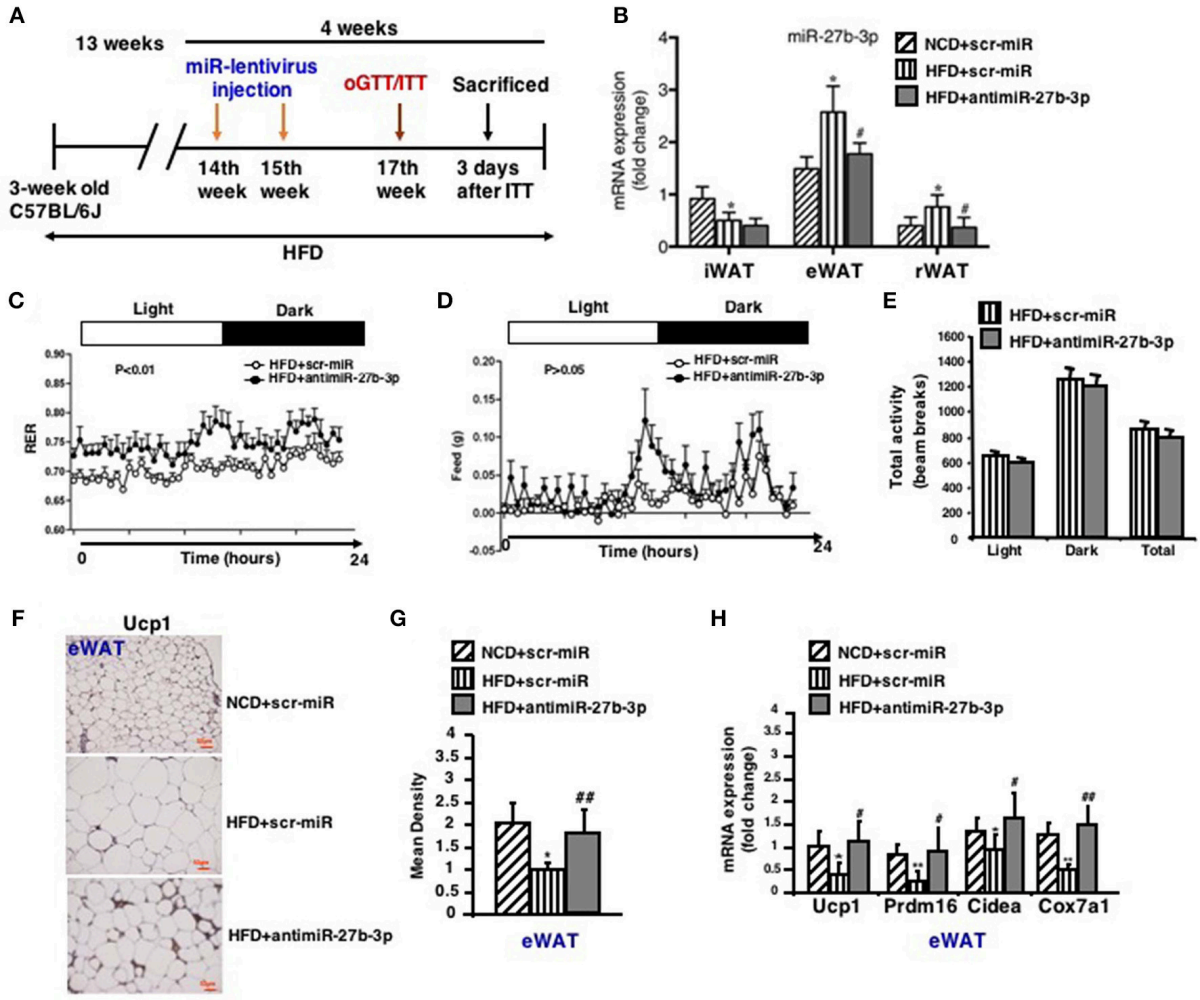
Inhibition of miR-27b-3p improves browning ability of eWAT. Mice were fed a HFD beginning at 3-week of age. Thirteen weeks later, mice received 2 injections of antimiR-27b-3p lentiviruses or scramble lentiviruses through the tail vein over 14 days. **(A)** Overview of anti-miR-27b-3p lentiviruses delivery. **(B)** qRT-PCR analysis of miR-27b-3p expression in iWAT, eWAT, and rWAT after mice were sacrificed (*n* = 12–13). **(C–E)** Energy expenditure of the mice during a 24-h period is reported as RER **(C)**, food intake **(D)**, and physical activity **(E)** were measured synchronously (*n* = 6, performed after two injections of anti-miR-27b-3p lentiviruses). **(F)** Representative Ucp1 staining in eWAT of the mice (*n* = 6). Scale bars: 50 μm. **(G)** Representative Ucp1 staining intensity designated by mean densitometry of the digital image (*n* = 6). **(H)** qRT-PCR analysis of Ucp1 and other brown adipose-selective genes in eWAT of the mice (*n* = 12–13). Data are shown as mean ± SEM. ^*^p < 0.05; ^**^p < 0.01, NCD+scr-miR vs. HFD+scr-miR; ^#^*p* < 0.05; ^##^*p* < 0.01, HFD+scr-miR vs. HFD+antimiR-27b-3p.

Based on the opposite trend of miR-27b-3p and Ucp1 expression during the course of HFD, we then considered whether miR-27b-3p is responsible for the decreased browning ability of WAT in mice fed an HFD. Thus, we tested to determine whether miR-27b-3p inhibition in HFD feeding mice could rescue browning ability of WAT. To this end, mice fed an HFD for 13 weeks were injected with lentiviruses encoding the antisense to miR-27b (anti-miR-27b) or scrambled control (scr-miR) through the tail vein to inhibit miR-27b-3p expression (Figure 4A). Initially, we verified the inhibition efficiency of miR-27b-3p. As mentioned above, an HFD led to increased levels of miR-27b-3p in eWAT and rWAT, but not in iWAT. Expression of miR-27b-3p in these three fat depots was suppressed by administration of antimiR-27b-3p lentiviruses (Figure 4B). We did whole body-energy metabolism analysis and observed a higher respiratory exchange rate (RER) during both the day and night cycles in the mice injected with antimiR-27b-3p lentivirus (Figure 4C). Total physical activity and food intake were both comparable between the two groups (Figures 4D,E). Other data on body-energy metabolism such as VO_2_ (ml/kg/h), VCO_2_ (ml/kg/h) and heat (kcal/h) were showed in Figure S3. Ucp1-expressing fat cells in eWAT were readily detected in the antimiR-27b-3p mice (Figures 4F,G). High-fat-fed antimiR-27b-3p mice expressed higher levels of brown fat-selected genes, such as Ucp1, Prdm16, Cidea, and Cox7a1 (Figure 4H). Although expression of Ucp1 mRNA and protein was also detected in iWAT and rWAT, the levels were not altered due to antimiR-27b-3p treatment (Figure S4). Taken together, these findings suggest antimiR-27b-induced browning effect in eWAT.

### Inhibition of miR-27b-3p Improves Metabolism of eWAT

**Figure 5:**
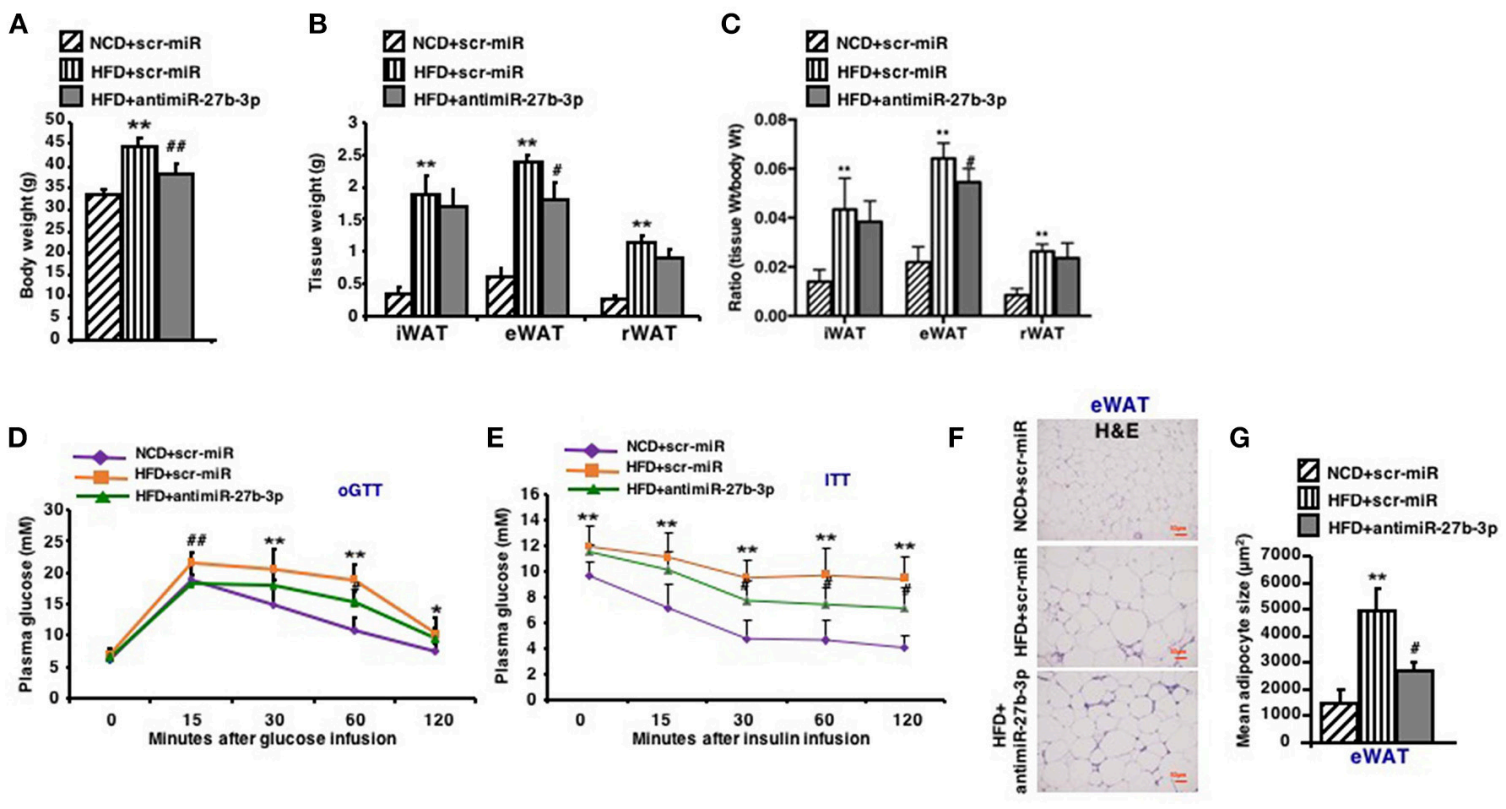
Inhibition of miR-27b-3p improves metabolism of eWAT. Mice were fed a HFD beginning at 3-week of age. Thirteen weeks later, mice received 2 injections of antimiR-27b-3p lentiviruses or scramble lentiviruses through the tail vein over 14 days. **(A)** Total body weight of mice (*n* = 12–13). **(B)** Tissue weight of iWAT, eWAT, and rWAT (*n* = 12–13). **(C)** Ratio of tissue weight to body weight (*n* = 12–13). **(D)** oGTT, performed at 17th week and mice were fasted for 16 h prior to obtain baseline blood glucose reading (*n* = 6). **(E)** ITT, performed at 17th week and mice were fasted for 6 h prior to obtain baseline blood glucose reading (*n* = 6). **(F)** Representative H&E staining in eWAT of the mice (*n* = 6). Scale bars: 50 μm. **(G)** Mean adipocyte size was measured under microscope (*n* = 6). Data are shown as mean ± SEM. ^*^p < 0.05; ^**^p < 0.01, NCD+scr-miR vs. HFD+scr-miR; ^#^*p* < 0.05; ^##^*p* < 0.01, HFD+scr-miR vs. HFD+antimiR-27b-3p.

In light of the effect of antimiR-27b-3p on browning ability of eWAT, high-fat-fed mice were subjected to metabolic analyses in response to lentiviruses injections of both scrambled control (scr-miR) and antimiR-27b-3p sequences. After 2 injections over 4 weeks, antimiR-27b-3p group gained less weight than the control group despite of HFD feeding (Figure 5A). Although the weight of all three fat depots increased due to high-fat feeding, mice treated with antimiR-27b-3p showed a dominant decrease in eWAT weight, but not in iWAT and rWAT (Figure 5B). The ratio of tissue weight to body weight showed the same results (Figure 5C). According to the result of whole body-energy metabolism analysis in Figure 4D, the suppressed weight gain was not associated with decreased food intake. Diet induced obesity is frequently associated with glucose intolerance. Notably, glucose intolerance was improved after the obese mice were injected with antimiR-27b-3p lentiviruses (Figure 5D). High-fat-fed mice treated with antimiR-27b-3p were also more insulin sensitive, as determined by insulin tolerance tests (Figure 5E). Furthermore, treatment of antimiR-27b-3p also led to decreased adipocyte cell size in eWAT (Figures 5F,G). Together, these data demonstrate that inhibition of miR-27b-3p counteracts weight gain and drive glucose disposal. These actions appear to occur mainly in the eWAT. It suggests that antimiR-27b-3p mice may be protected from obesity on a high-fat diet because of a significant increase in energy expenditure that is not linked to physical activity.

### miR-27b-3p Inhibition Enhances Browning Ability of Adipocytes From BAT

**Figure 6:**
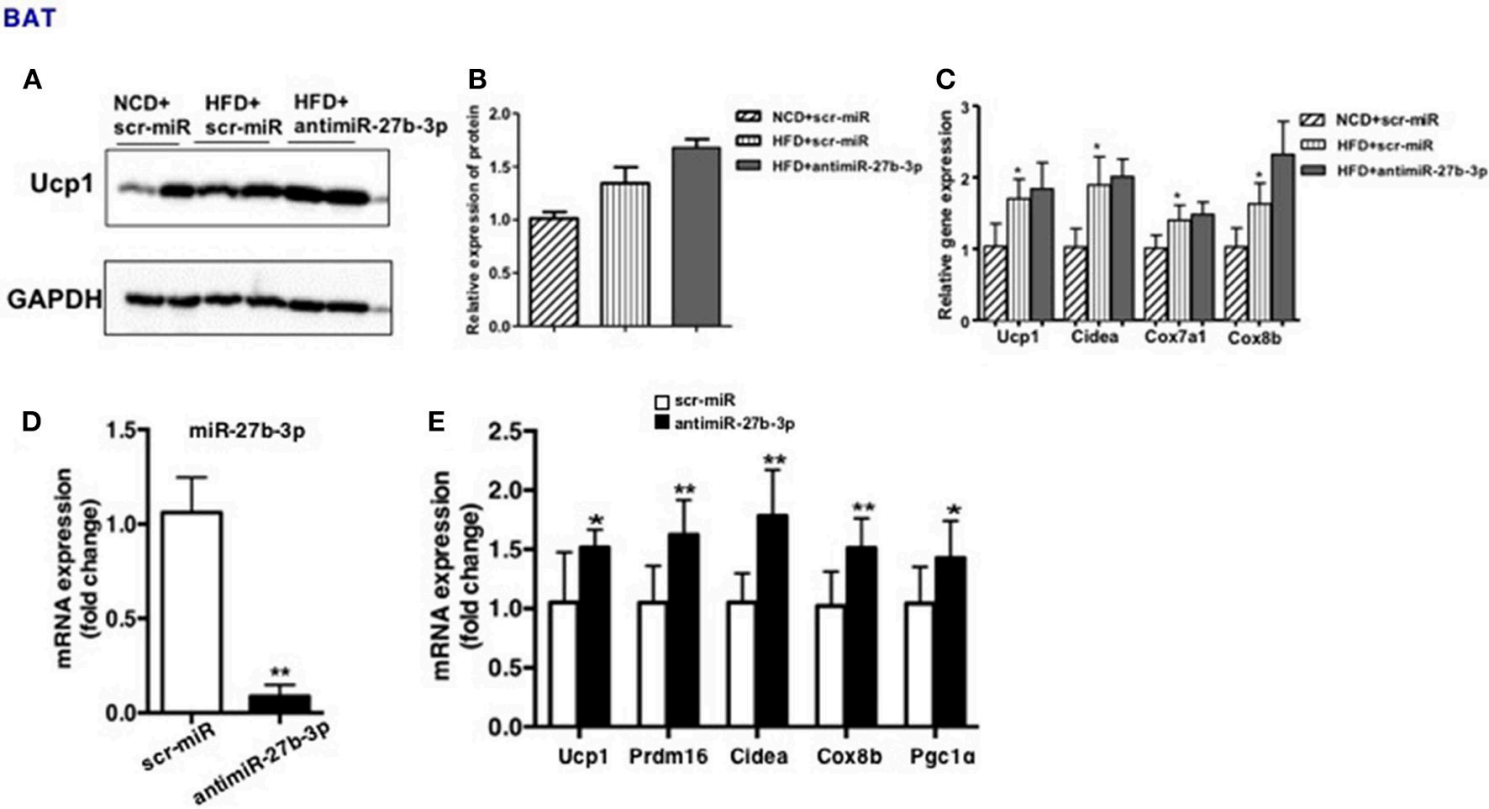
miR-27b-3p inhibition enhances browning ability of adipocytes from BAT. **(A–C)** Mice were fed a HFD beginning at 3-week of age. Thirteen weeks later, mice received 2 injections of antimiR-27b-3p lentiviruses or scramble lentiviruses through the tail vein over 14 days. **(A)** Western blot analysis of Ucp1 expression in BAT of the mice. **(B)** Quantitative analysis of Ucp1 was performed with densitometric image analysis software (*n* = 6). **(C)** qRT-PCR analysis of Ucp1 and other brown adipose-selective genes expression in BAT of the mice. **(D,E)** SVF cells from BAT was isolated from 3-week-old male C57BL/6J mice. **(D)** qRT-PCR analysis of miR-27b-3p expression. **(E)** qRT-PCR analysis of Ucp1 and other brown adipose-selective genes: Prdm16, Cidea, Cox8b and Pgc1α. Data are presented as mean ± SEM; *n* = 3; ^*^p < 0.05; ^**^p < 0.01.

Due to systemic treatment with lentivirus, the effect of miR-27b-3p inhibition on development and physiology of BAT or skeletal muscle cannot be excluded. To determine if miR-27b-3p inhibition *in vivo* could enhance browning ability of BAT, we performed qRT-PCR and western blotting to analyze BAT function after miR-27b-3p inhibition. We found that high-fat diet accelerated mice's brown fat function modestly, such as Ucp1, Cidea, Cox7a1, and Cox8b, while anti-miR-27b-3p had only slight effect on BAT function on HFD mice without statistical difference (Figure 6C). Consistent with qRT-PCR, western blotting also showed that Ucp1 expression in BAT increased slightly in HFD mice and there were no significant change with HFD mice after anti-miR-27b inhibition (Figures 6A,B). Thus, miR-27b-3p inhibition *in vivo* had no effect on BAT. Skeletal muscle was not assessed in this study.

Moreover, a direct effect of miR-27b-3p inhibition on brown adipocytes had been demonstrated. Primary stromal vascular fraction (SVF) cells from BAT were cultured and transfected with antimiR-27b-3p oligonucleotides (Figure 6D), and then differentiated to mature. The results displayed that after miR-27b-3p inhibition, expression of Ucp1 and other brown adipose-selective genes: Prdm16, Cidea, Cox8b, and Pgc1α of adipocytes from BAT increased (Figure 6E). These data suggest that different from the data *in vivo*, miR-27b-3p inhibition has direct effect on browning of adipocytes from BAT.

## Discussion

It is well-known that different fat compartments are associated with differential metabolic risk (15–17). In particular, VAT has traditionally been considered a highly pathogenic adipose tissue compartment compared with SAT. Abnormally high deposition of VAT, also known as visceral obesity, is associated with poor prognosis, metabolic disturbances, and increased degree of pathology in several chronic diseases (18, 19). Therefore, there is a great urgency to reduce VAT accumulation. Since previous studies have concentrated on SAT when focusing on browning ability of WAT, less reports were seen about VAT browning. As early as in 2010, Petrovic et al. (20) had found that epididymally derived white adipocyte expressed Ucp1 via stimulation of rosiglitazone, one kind of peroxisome proliferator-activated receptor γ agonists and suggested these cells had genuine thermogenic capacity. Recent study also showed that selective genetic ablation of Zfp423, a transcriptional suppressor of the adipocyte thermogenic program, resulted in accumulation of beige-like thermogenic adipocytes within multiple visceral adipose depots (21). In our present study, we find that inhibition of miR-27b-3p could enhance browning capacity of mice epididymal fat, decrease eWAT accumulation and improve insulin sensitivity.

To date, the molecular mechanisms governing the browning of VAT are incompletely understood. Now it is well-known that miRNAs play pivotal roles in numerous biological processes of adipose tissues. The miR-27 family is one of the first to be identified and functionally links to white adipogenesis (22–26). Moreover, several studies also have revealed that miR-27 is involved in the regulation of brown and beige adipogenesis and the thermogenic program. Kang et al. (27) found that miR-27 negatively regulated adipogenesis and impairing mitochondrial biogenesis, structure, and activity by targeting prohibitin. Inhibition of miR-27 in brown preadipocytes enhanced the expression of multiple key transcription factors including Pparα, Pparγ, Prdm16, and Ppargc1α, and facilitated the differentiation toward Ucp1 expressing adipocytes (28). In our previous study, we also have demonstrated that levels of miR-27b-3p in human VAT and serum correlate positively with body mass index (BMI) and waist-hip ratio (WHR), and miR-27b-3p inhibition leads to enhanced browning ability in visceral adipocytes (14). In this study, we find the highest expression of miR-27b-3p in eWAT compared with iWAT and rWAT. Moreover, we also validate higher expression of miR-27b-3p in eWAT of mice fed an HFD than in control mice and miR-27b-3p inhibition *in vivo* boosts browning capacity of mice epididymal fat and improve insulin sensitivity. Consistent with the significant upregulation of miR-27b-3p at the 4th week of high-fat feeding, expression of Ucp1, the marker protein of fat browning is downregulated meanwhile. Thus, high level of miR-27b-3p in VAT may be the cause of low browning capacity of visceral fat.

Besides the roles in the regulation of adipocyte differentiation and obesity, miR-27 family also play important role in inflammatory response and immune response through PPARs and RXRα (29, 30) or NF-κB pathway (31). Moreover, miR-27b may function as a tumor suppressor in neuroblastoma (32) and colorectal cancer (33). Considering the key roles of miRNAs and as a therapeutic tool, delivering anti-miRNA compounds into the body has the potential to become a new class of drugs (34–36). Small size and conserved sequence of miRNAs makes them become attractive candidates from a developmental standpoint (37–40). In our current research, we have delivered antimiR-27b-3p into the mice through tail vein injection in order to enhance browning ability of WAT and improve insulin sensitivity. Not like our previous study that in mice treated with glucocorticoids, SAT is the most affected adipose tissue by antimiR-27b-3p, in mice fed an HFD, VAT browning is enhanced via antimiR-27b-3p injection. The potential mechanisms may relate to the highest level of miR-27b-3p in VAT and that an HFD does not lead to increasing expression of miR-27b-3p in SAT.

Taken together, our findings highlight the decreased browning ability of eWAT in obese mice and the role of miR-27b-3p in regulating browning of eWAT in mice with high-fat feeding. It suggests that miR-27b-3p should be further explored as a potential target for the treatment of central obesity and its accompanying diseases.

## Author Contributions

JY, YL, and FW performed experiments and participated in the discussion and writing of the manuscript. XK and WD contributed to the lentiviral production and further tail vein injection experiments. JL contributed to the whole-body energy metabolism experiments. YS and SL contributed to the oGTT and ITT experiments. GD contributed to the project concept and experimental design. GD is the guarantor of this work and, as such, had full access to all the data in the study and takes responsibility for the integrity of the data and the accuracy of the data analysis.

### Conflict of Interest Statement

The authors declare that the research was conducted in the absence of any commercial or financial relationships that could be construed as a potential conflict of interest.
