# Supplementary material for: MiR-27b-3p Inhibition Enhances Browning of Epididymal Fat in High-Fat Diet Induced Obese Mice
Source: Front Endocrinol (Lausanne). 2019 Feb 4;10:38. doi: 10.3389/fendo.2019.00038 (PMC6369196; doi:10.3389/fendo.2019.00038)
Supplement: Supplementary file 1 [file Data_Sheet_1.docx]

***Supplementary Material***

**MiR-27b-3p Inhibition Enhances Browning of Epididymal Fat in High-fat Diet Induced Obese Mice**

**Jing Yu, Yifan Lv, Fengliang Wang, Xiaocen Kong, Wenjuan Di, Juan Liu, Yunlu Sheng, Shan Lv, Guoxian Ding^*^**

*** Correspondence:** Guoxian Ding: dinggx@njmu.edu.cn

**Supplementary Table**

Table S1. PCR Oligonucleotide Primers

| β-actin | forward | 5’-GGCTGTATTCCCCTCCATCG-3’ |
| --- | --- | --- |
|  | reverse | 5’-CCAGTTGGTAACAATGCCATGT-3’ |
| Ucp1 | forward | 5’-AGGCTTCCAGTACCATTAGGT-3’ |
|  | reverse | 5’-CTGAGTGAGGCAAAGCTGATTT-3’ |
| Cidea | forward | 5’-TGACATTCATGGGATTGCAGAC-3’ |
|  | reverse | 5’-GGCCAGTTGTGATGACTAAGAC-3’ |
| Prdm16 | forward | 5’- CCACCAGCGAGGACTTCAC -3’ |
|  | reverse | 5’- GGAGGACTCTCGTAGCTCGAA -3’ |
| Cox7a1 | forward | 5’- GCTCTGGTCCGGTCTTTTAGC -3’ |
|  | reverse | 5’- GTACTGGGAGGTCATTGTCGG -3’ |
| Pgc1α | forward | 5’- TATGGAGTGACATAGAGTGTGCT -3’ |
|  | reverse | 5’- CCACTTCAATCCACCCAGAAAG -3’ |
| Adipoq | forward | 5’- TGTTCCTCTTAATCCTGCCCA -3’ |
|  | reverse | 5’- CCAACCTGCACAAGTTCCCTT -3’ |
| Visfatin | forward | 5’- CCCGATTGAAGTAAAGGCTGT -3’ |
|  | reverse | 5’- TGGTAAGCCAGTAGCACTCTG -3’ |
| Leptin | forward | 5’- GTGGCTTTGGTCCTATCTGTC -3’ |
|  | reverse | 5’- CGTGTGTGAAATGTCATTGATCC -3’ |
| Resistin | forward | 5’- AAGAACCTTTCATTTCCCCTCCT -3’ |
|  | reverse | 5’- GTCCAGCAATTTAAGCCAATGTT -3’ |
| MCP-1 | forward | 5’- TAGGTGAGAGATGCAGCACG-3’ |
|  | reverse | 5’- GTCTTTGCAGAACGTGCCAG-3’ |

| **miRNAs** |  | **5’-3’** |
| --- | --- | --- |
| Rnu6 | Sequence: | GTGCTCGCTTCGGCAGCACATATACTAAAATTGGAACGATACAGAGAAGATTAGCATGGCCCCTGCGCAAGGATGACACGCAAATTCGTGAAGCGTTCCATATTTT |
|  | Reverse: | GTCGTATCCAGTGCAGGGTCCGAGGTATTCGCACTGGATACGACAAAATA |
|  | Upstream:  Downstream： | CTCGCTTCGGCAGCACATA |
|  |  | GTGCAGGGTCCGAGGT |
| miR-27b-3p | Sequence: | UUCACAGUGGCUAAGUUCUGC |
|  | Reverse: | GTCGTATCCAGTGCAGGGTCCGAGGTATTCGCACTGGATACGACGCAGAA |
|  | Upstream:  Downstream: | CGCGTTCACAGTGGCTAAG |
|  |  | GTGCAGGGTCCGAGGT |

**Supplementary Figures**

**
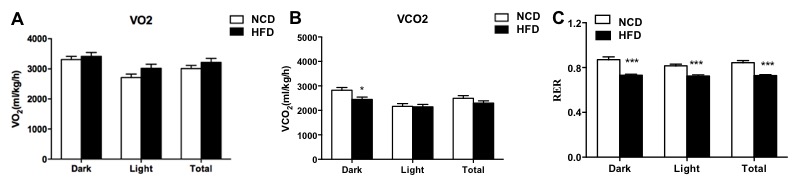
**

**Figure S1. Energy expenditure of the mice fed an HFD.** **(A)** Oxygen consumption of mice with HFD during the metabolic cage measurement. **(B)** Carbon dioxide production from mice with HFD during the metabolic cage measurement. **(C)** RER of mice that have been fed with HFD during the metabolic cage measurement (n = 6). Data are shown as mean ± SEM. **p*<0.05; ****p*<0.001.

**
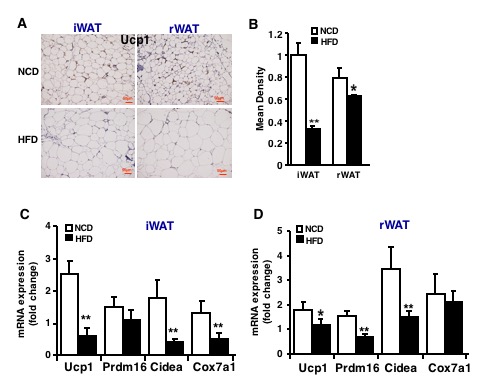
**

**Figure S2. Ucp1 expression in iWAT and rWAT of HFD induced obese mice. (A)** Representative immunohistochemical staining for Ucp1 in iWAT and rWAT of HFD induce obese mice and the control (n=6). Scale bars: 50μm. **(B)** Representative Ucp1 staining intensity designated by mean densitometry of the digital image (n=6). **(C-D)** qRT-PCR analysis of Ucp1 and other brown adipose-selective genes in iWAT and rWAT of HFD induced obese mice and the control (n=10-12). Data are shown as mean ± SEM. **p*<0.05; ***p*<0.01.

**
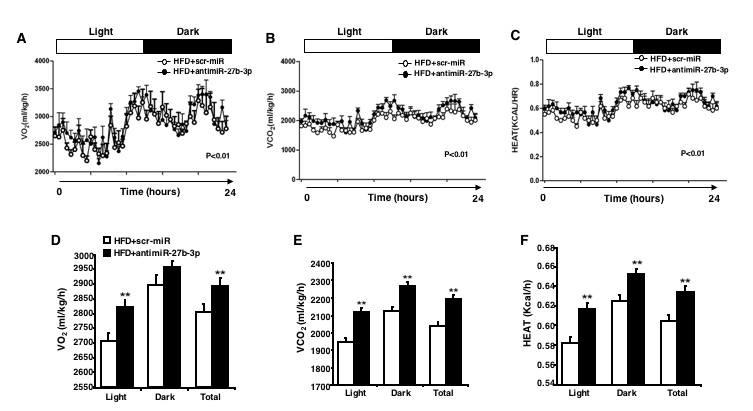
**

**Figure S3. Energy expenditure of the mice fed an HFD after two injections of anti-miR-27b-3p lentiviruses.** (**A** and **D**) VO2, (**B** and **E**) VCO2, (**C** and **F**) Heat. Data are shown as mean ± SEM, n=6, ***p*<0.01.


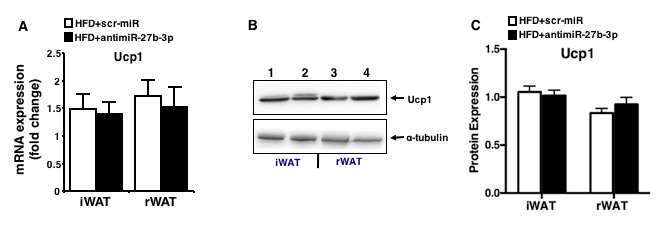


**Figure S4. Ucp1 expression in iWAT and rWAT of obese mice after injected with anti-miR-27b-3p lentiviruses. (A)** qRT-PCR analysis of Ucp1 expression in iWAT and rWAT of mice fed HFD and then injected with two injections of anti-miR-27b-3p lentiviruses (n=12-13). **(B)** Western blot analysis of Ucp1 expression in iWAT and rWAT of the mice. **(C)** Quantitative analysis of Ucp1 was performed with densitometric image analysis software (n=6). Data are shown as mean ± SEM.
